# *In vivo* 13C-MRI using SAMBADENA

**DOI:** 10.1371/journal.pone.0200141

**Published:** 2018-07-12

**Authors:** Andreas B. Schmidt, Stephan Berner, Moritz Braig, Mirko Zimmermann, Jürgen Hennig, Dominik von Elverfeldt, Jan-Bernd Hövener

**Affiliations:** 1 Medical Physics, Department of Radiology, Medical Center–University of Freiburg, Faculty of Medicine, University of Freiburg, Freiburg, Germany; 2 Section Biomedical Imaging, MOIN CC, Department of Radiology and Neuroradiology, University Medical Center, University of Kiel, Kiel, Germany; 3 German Consortium for Cancer Research (DKTK), Heidelberg, Germany; 4 German Cancer Research Center (DKFZ), Heidelberg, Germany; Martin-Luther-Universitat Halle-Wittenberg, GERMANY

## Abstract

Magnetic Resonance Imaging (MRI) is a powerful imaging tool but suffers from a low sensitivity that severely limits its use for detecting metabolism *in vivo*. Hyperpolarization (HP) methods have demonstrated MRI signal enhancement by several orders of magnitude, enabling the detection of metabolism with a sensitivity that was hitherto inaccessible. While it holds great promise, HP is typically relatively slow (hours), expensive (million $, €) and requires a dedicated device (“polarizer”). Recently, we introduced a new method that creates HP tracers without an external polarizer but within the MR-system itself based on *para*hydrogen induced polarization (PHIP): Synthesis Amid the Magnet Bore Allows Dramatically Enhanced Nuclear Alignment (SAMBADENA). To date, this method is the simplest and least cost-intensive method for hyperpolarized ^13^C-MRI. HP of *P*_13C_ > 20% was demonstrated for 5mM tracer solutions previously. Here, we present a setup and procedure that enabled the first *in vivo* application of SAMBADENA: Within seconds, a hyperpolarized angiography tracer was produced and injected into an adult mouse. Subsequently, fast ^13^C-MRI was acquired which exhibited the vena cava, aorta and femoral arteries of the rodent. This first SAMBADENA *in vivo* ^13^C-angiography demonstrates the potential of the method as a fast, simple, low-cost alternative to produce HP-tracers to unlock the vast but hidden powers of MRI.

## Introduction

Within the last decades, Magnetic Resonance (MR) has become a powerful tool for human healthcare and chemical analysis [1–6]. However, the effect that gives rise to MR is small and as a result the method itself is insensitive: Of all MR-active nuclei, only the by-far most abundant element with the strongest magnetic moment of all stable nuclei, ^1^H, provides sufficient signal for clinical routine. Worse still: the allegedly most interesting information for diagnostics that is contained in the MR signal, the spectroscopic fingerprints of molecules, is–with few exceptions–too weak for routine clinical use [7,8]. As a result, of all the rich information contained in the MR signal, typically, only the spatial distribution, relaxation properties and motion of protons is resolved by modern MRI.

However, it is well known that there is a huge potential to improve the sensitivity of MRI: because of the low thermal polarization, effectively, no more than a few parts per million of all spins contribute to the MR signal. The hyperpolarization (HP) of nuclear spins uses this reservoir to increase the sensitivity of MRI by several orders of magnitude. The enhanced MR signal of hyperpolarized agents has enabled to monitor metabolism non-invasively and *in vivo* in both, preclinical studies and clinical trials [8–11]. Moreover, some hyperpolarized molecules were used to map/image pH-value, which is interesting e.g. for oncology [12,13].

With few exceptions, these HP agents were produced in an external polarizer based on dissolution dynamic nuclear polarization (d-DNP). While d-DNP is versatile and has enabled most of the progress towards a diagnostic application of HP it is, to date, relatively complex and costly. Another issue shared by all polarization methods is that some of the precious, short-lived enhancement of the HP-tracer is inevitably lost during the transfer from the polarizer into the MRI system, although storage at cold temperature may improve this matter [14].

To address these challenges, a new, simple method was recently introduced that enables the production of HP tracers within the MR-system itself. By synthesis amidst the magnet bore, a dramatically enhanced nuclear alignment (SAMBADENA) [15] was obtained—an extra polarizer or sample transfer was no longer required. This *para*hydrogen-induced polarization (PHIP) method achieved a ^13^C-polarization of *P* = (21 ± 2)%, corresponding to enhancement factor of the ^13^C-signal of *η* ≈ 35.000 at a magnetic field of *B*_0_ = 7 T (angiography tracer hydroxyethyl propionate (HEP), concentration of *c* = 5 mM, temperature of T ≈ 80°C, pressure of *p* = 15 bar; data reported in Ref. [15]). The HP was generated at high field under so-called PASADENA conditions [16].

Whereas these were promising first results, no *in vivo* application was demonstrated yet. This, along with the HP of metabolically interesting agents and catalyst removal, are the current challenges for the method. Note, though, that there are quite interesting advances reported recently regarding metabolically active tracers [17–25] and catalyst removal [26–29].

For an *in vivo* application of SAMBADENA, the following challenges arise:

- the constraints imposed on the HP-production by the confined space, magnetic field, required reaction temperature and reaction pressure in the bore of the MRI,
- the tracer solution must be sterile, at a suitable temperature and pressure before the injection,
- the solvent must be biocompatible
- the injection must be performed well-controlled, and
- a high concentration of the tracer is required to provide sufficient SNR *in vivo*,
- furthermore, though not addressed here, the rhodium-based hydrogenation catalyst must be removed from the sample and a biocompatible tracer must be selected, at least for potential human studies.

In this contribution, we address these challenges and present an approach which allows production of hyperpolarized agents, subsequent *in vivo* application and imaging within seconds.

## Materials and methods

The *para-*hydrogen (*p*H_2_) fraction of dihydrogen gas (H_2_ purity of ≥ 99,999%) was enriched to > 90%, filled into aluminum cylinders and used on demand as described previously [30].

### Setup

A 7T preclinical MRI system (Biospec 7/20, PV5.1, Bruker, Germany) and a dual-resonant ^1^H-^13^C transmit-receive volume coil (length of 10 cm, diameter of 7.2 cm, Rapid, Germany) were used for MRI and HP (Setup 1). In some MRI experiments, a ^13^C-receive surface coil (Rapid, Germany) was added to acquire signal with increased sensitivity (Setup 2). Note that here-reported *in vivo* data was measured before Setup 2 was implemented using Setup 1.

**Fig 1:**
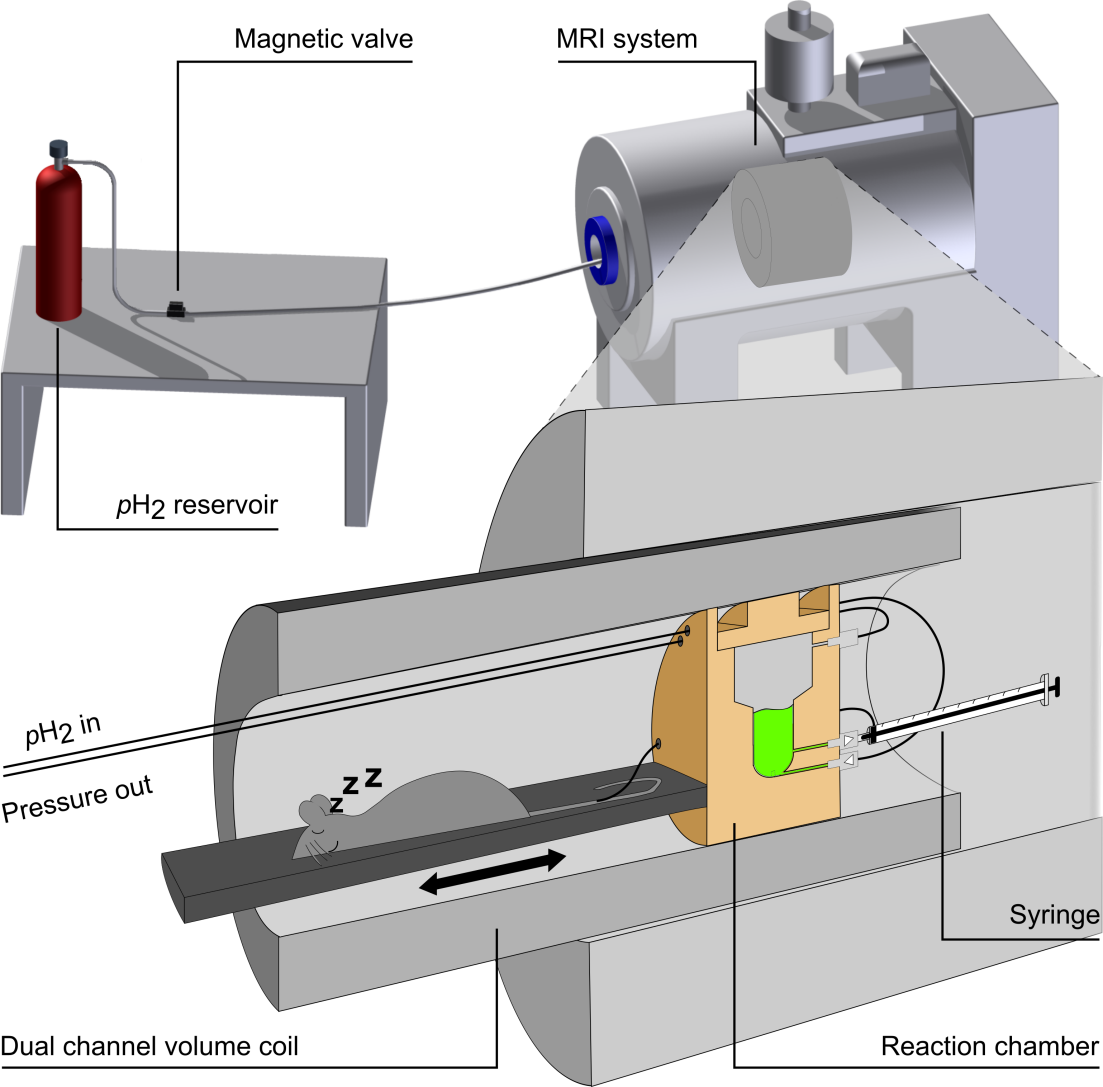
Schematic view of the setup used for *in vivo* MRI with hyperpolarized agents. The HP contrast agent (green) was prepared in a reaction chamber (yellow) at the isocenter of the MRI scanner, next to the animal. After the HP, the agent was ejected from the reactor into a syringe for injection *in vivo*. At the same time, the setup was moved such that the animal was in the isocenter and ^13^C-MRI was carried out (see Fig 2). Note that the entire setup consists of a *p*H_2_ reservoir, two magnetic valves, two one-way valves, the reactor, an animal bed, a syringe, a catheter, a cannula, a ^1^H-^13^C volume resonator and a dual-channel MRI system. Figure adapted with permission from [15] under creative commons license CC BY 4.0 (https://creativecommons.org/licenses/by/4.0).

**Fig 2:**
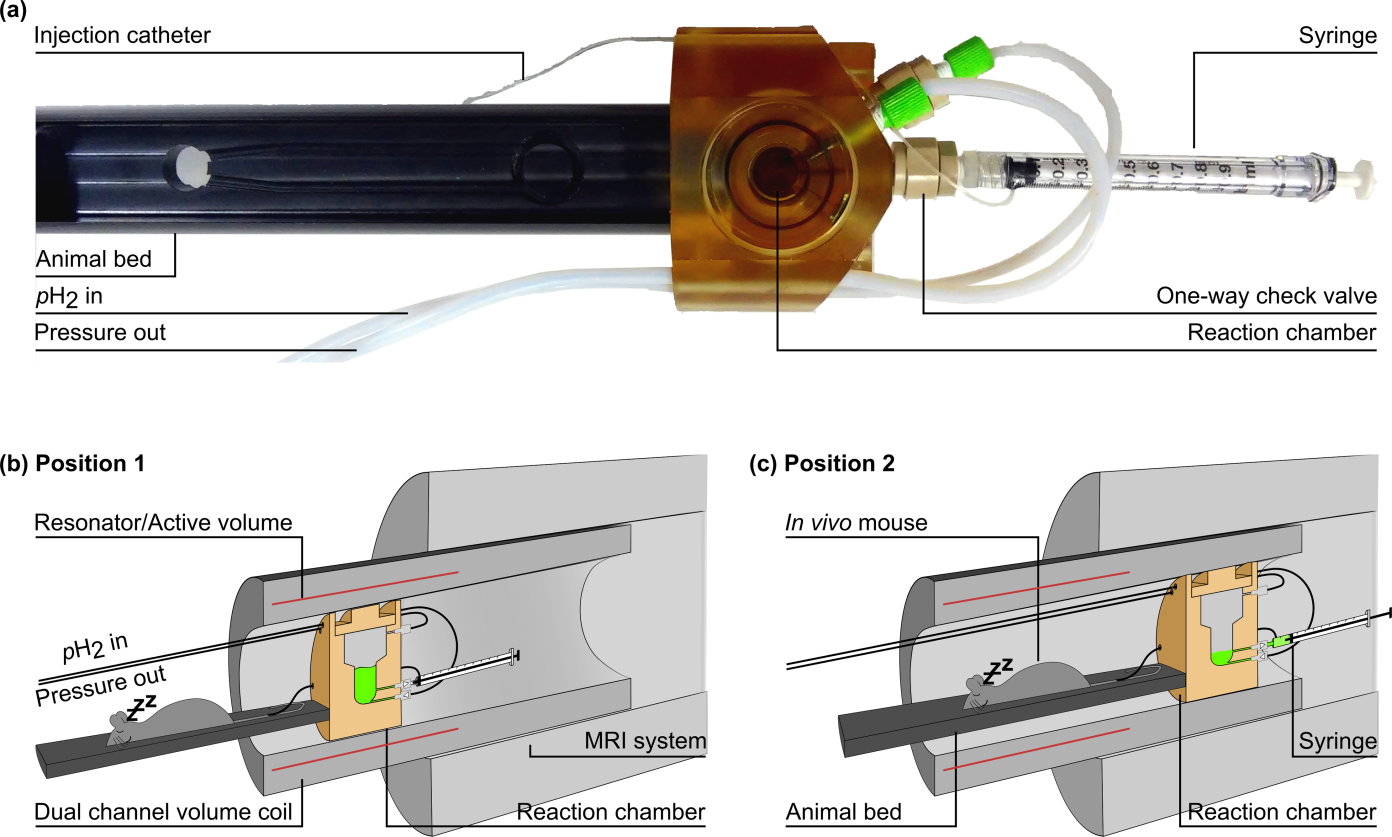
Picture and schematic view of the setup that allowed hyperpolarization and imaging within seconds. The SAMBADENA reactor was combined with a custom-built mouse bed and mounted to a motorized slider. The mouse bed allowed monitoring, heating and anesthesia of the animal (a). For an *in vivo* experiment, HP was performed at ~80°C and 15 bar with the reactor in the isocenter (Position 1, b). Subsequently, the pressure was released from the reactor, the temperature was adjusted to (35 ± 1)°C and the agent was ejected into a syringe. The setup was moved using the motorized slider until the animal was in the isocenter (Position 2, c). Simultaneously, manual injection was performed. Note that different shim settings that were obtained before were used for the two positions. About 15 s after HP, imaging was commenced.

A high pressure and high temperature reaction chamber was custom made from polysulfone to yield fast and effective hydrogenation (inner volume of ≈ 2 ml, PSU 1000) (Fig 1). The reactor was combined with a custom-made animal bed that allowed preparation, anesthesia and monitoring the animal (Fig 1 and Fig 2).

Outside of the MRI, two magnetic valves (V1, V2, type 0124, Bürkert, Germany) were used to inject and release pressurized *p*H_2_ from the reactor. The valves were controlled and synchronized with the MRI using the digital outputs of a data acquisition board (DAQ 6125, National Instruments, USA) and custom-written software (MATLAB R2012b, MathWorks, USA).

The **precursor solution** was prepared from 1-^13^C- and 2,3,3-^2^H-labeled hydroxyethyl-acrylate (HEA) [8–10] and a catalyst in deionized H_2_O. By addition of *p*H_2_, hydroxyethyl-propionate was formed [31,32] (HEP, longitudinal ^13^C relaxation time *T*_1_ of (75 ± 5) s *in vitro*, in H_2_O, at 7 T [15]). HEP was selected because it was hyperpolarized at high magnetic fields before [15], in contrast to other established PHIP-molecules like succinate.

The catalyst was formed by a biphosphine ligand (1,4-bis-[(phenyl-3-propane sulfonate) phosphine] butane disodium salt, MW = 562.53 g·mol^-1^, MDL number MFCD15144866, Sigma Aldrich, MO, USA) and a rhodium complex (bis(norbornadiene)rhodium (I) tetrafluoroborate, MW = 373.99 g·mol^-1^, hazard statement H314, CAS 36620-11-8, StremChemicals, MA,USA) [15,33,34]. The ligand was added in 10% excess compared to the rhodium complex.

### Spin order transfer (SOT)

The *l*-PH-INEPT+ [35,36] sequence was used to transform ^1^H singlet spin order into ^13^C-HP. The sequence consists of four pulses (45°y ^1^H – 90°y ^1^H and 90°x ^13^C – 90°x ^13^C) and two free-evolution intervals *t*_1_ and *t*_2_ interleaved with 180° refocusing pulses on ^1^H and ^13^C. The duration of the free-evolution intervals were optimized with respect to polarization yield by means of quantum mechanical simulation (*t*_1_ = 69.8 ms and *t*_2_ = 38.9 ms, total 108.7 ms) [15,36].

### Hyperpolarization

Hot precursor solution was filled into the heated reactor (both ≈ 80°C). The reactor was connected to an animal bed and placed in the isocenter of the magnet. The hydrogenation was initiated by injecting pressurized *p*H_2_ (≈ 15 bar) from below. Depending on the concentration of the tracer of 5 mM or 80 mM a hydrogenation time of *t*_h_ = 5 s or 8 s was applied, respectively. Subsequently, the SOT sequence was executed and longitudinal ^13^C polarization created (total time of SOT of 108.7 ms). In total, one injection dose of an 80 mM HP agent was produced within the MRI, next to the animal, in 8s.

*In vitro* quantification of the polarization was achieved by comparing the signal of a hyperpolarized sample to that of a thermally polarized model solution acquired in a separate scan (5 ml n.a. acetone in a test tube, 748 mmol ^13^C, *T*_1_ ≈ 10 s), using the same acquisition parameters. 100% hydrogenation of the agent and 100% *p*H_2_ were assumed.

It was previously shown that this assumption is not correct and results in an underestimation of HP of the hydrogenated fraction. In other words, hydrogenation was not fully completed after the short hydrogenation time of 8s but a trade-off between hydrogenation and relaxation of the singlet order was made [15]. The quantification of HP and incomplete hydrogenation are discussed in more detail in S1 Text.

### Injection of the tracer

The injection of the tracer solution was realized by a setup custom build for operation inside the 20-cm bore of the MR system at 7T. To optimize the procedure and MRI, a test object (phantom) was manufactured (S4 Fig): A thin tube (inner diameter of 1 mm) was wound around a 1 ml syringe and placed inside a 15 ml tube (CELLSTAR® Polypropylene Tube, Greiner, Austria) filled with water. The end of the hose was connected to the tip of the syringe.

After the production of an HP tracer, the pressure was reduced from 15 bar to close to ambient pressure in ~2 s by shortly opening and closing valve V2 several times (open for 100 ms–200 ms–400 ms–800 ms, separated by intervals of 200 ms where V2 was closed). Subsequently, the HP solution was manually extracted into a polycarbonate syringe inside of the magnet (1 ml, Luer-Lok, BD, Germany, at room temperature). The syringe was connected to the bottom of the reaction chamber via an in-line one-way valve (IDEX, USA). Additionally, a thin catheter was added to the syringe to guide the tracer solution into the phantom or the animal via tail vein (Fig 2). About 5 s after HP, the tracer was injected manually over a period of 5–10 s. During this time, assuming a *T*_1_ of 75 s (HEP in H_2_O) and an initial HP of 4.9%, polarization decreased to approximately 4.0%. Air bubbles in the injection path were avoided by filling it with ~70–80 μl of saline solution prior to experiments. The injection was tested several times without the animal outside of the magnet, and no bubbles were observed in the catheter or syringe.

### *In vivo* experiment

The study was carried out in compliance with internationally accepted recommendations and guidelines for handling of experimental animals. The local animal ethics committee has specifically approved this study (Referat 35, Regierungspraesidium Freiburg, Bertoldstr. 43, 79098 Freiburg; AZ:35–9185.81/G-14/91). A C57BL/6N mouse was used, a common inbred strain for animal research with no undertaken genetic modifications (~30 g; 14 ± 4 weeks).

Anesthesia was performed using isoflurane (1–2% in >99.5% O_2_, ~1.2 L·min^-1^, during spontaneous breathing) and the animal physiology was continuously monitored using a monitoring device (SA Instruments 1030, Stony Brook, NY 11790) and adjusted throughout the experiment: The respiration rate was maintained at about 70 min^-1^ by adjusting the anesthesia depth. To detect respiration, a pressure sensitive cushion was positioned underneath the animal. Measured heart rates were between 410 to 600 bpm. Body temperature was maintained at about 36.4–37°C using a custom-made water circulation system at the bottom of the used animal bed that was driven by a water pump (1P T12184, Thermo Fisher Scientific, Newington, NH, USA). All efforts were made to minimize suffering, and the animal was sacrificed after the experiment.

### MRI

After HP, the animal bed was moved into the isocenter of the magnet using a motorized slider (Bruker, Germany). Note that two different shim settings for HP and MRI were obtained before and applied accordingly.

For the *in vivo* experiment, one hyperpolarized batch was used for two separate injections (each 150 μl). After each injection, imaging was performed (~15 s and 30 s after HP) using a single shot, ^13^C-RARE sequence [37] (90/180°, RARE-factor: 38, FOV: (8.4cm)^2^, acquisition matrix of 128x96 px, interpolated to 256x192 px, in-plane resolution: 0.33 x 0.44 mm, one slice with a thickness of 6 cm, *T*_R_ = 0.487 s, *T*_E_ = 79 ms, acquisition time: 0.487 s).

For anatomical reference, a multi-slice ^1^H-MRI was acquired after ^13^C-MRI (^1^H-Turbo-RARE, 90/180°, RARE-factor: 8, matrix: 256^2^, in-plane resolution: (0.33 mm)^2^, *T*_R_ = 2.5 s, *T*_E_ = 33 ms, acquisition time: 161 s). ^13^C SNR was quantified by dividing the highest signal by the standard deviation of the noise in an 1 cm^2^ region of interest (ROI), apparently signal-free region in the image (MATLAB R2012b, MathWorks, USA).

## Results

### Polarization yield

A highly concentrated HEP-tracer solution (80 mM HEA) was successfully polarized *in vitro* and quantified to *P* = (4.9 ± 0.6)%, assuming 100% *p*H_2_ fraction and hydrogenation (mean ± std. error of *N* = 3 experiments, 4 mM catalyst in H_2_O, *t*_h_ = 8 s; see S1 Fig). Full hydrogenation, however, was not achieved within the short time for hydrogenation. This is reflected in the finding that a much higher polarization, *P* = (21 ± 2)%, was obtained for a lower tracer concentration of 5 mM [15] (also see S1 Text). Note that in a previous study, *P* = (7 ± 1)% was achieved in D_2_O but under otherwise similar conditions (mean ± st.dev. of *N* = 3 experiments, 80mM HEA, 15 bar, ~80°C [15]). When HP of an 80 mM tracer solution was measured as function of the concentration of the catalyst in the range of 1 mM to 4 mM, no statistically relevant deviations were found (see S1 Fig)

### Achieving *in vivo* conditions

Prior to *in vivo* application, the hyperpolarized solution was adjusted to body temperature and ambient pressure. By releasing pressure as described above, fast pressure release was realized while losing < 100 μl of solution into the outlet path of the reactor.

The temperature of 600 μl solution was found to reduce from ~80°C to (35 ± 1)°C (mean ± st.dev.) during the process (~5 s). The change of temperature is attributed to heat exchange with the syringe and other components with high thermal conductivity and capacity of the injection unit. Thus, the method allowed to prepare an injection dose ready and suited for *in vivo* application within few seconds.

### Imaging phantom and reproducibility

150 μl of HP solution were produced and injected into the imaging phantom to evaluate the reproducibility for MRI (5 mM HEA, 1 mM catalyst in H_2_O, *t*_h_ = 5 s; note that concentration of the tracer was chosen 16x lower than 80 mM to simulate lower HP and concentration *in vivo*, where the tracer is diluted with blood to ~10 mM). ^13^C- and ^1^H-MRI were acquired. Superposition of the images revealed that the ^13^C signal was located in the small tube of the phantom (S4 Fig). The same experiment was repeated three times with 300 μl of HP solution (5 mM HEA, 1 mM catalyst in H_2_O, *t*_h_ = 5 s). SNR was determined for each scan at two different positions: using the maximum signal in the image (SNRmax) and using the signal at a 3 x 1 voxel ROI of the catheter (SNRcat; ROI selected from a piece of the catheter that was close to the surface coil but not overlaid with signal from other parts of the phantom). The reproducibility of the SNR obtained from these three experiments was SNRmax = (418 ± 72) and SNRcat = (110 ± 29) (mean ± st.dev.; S6 Fig).

**Fig 3:**
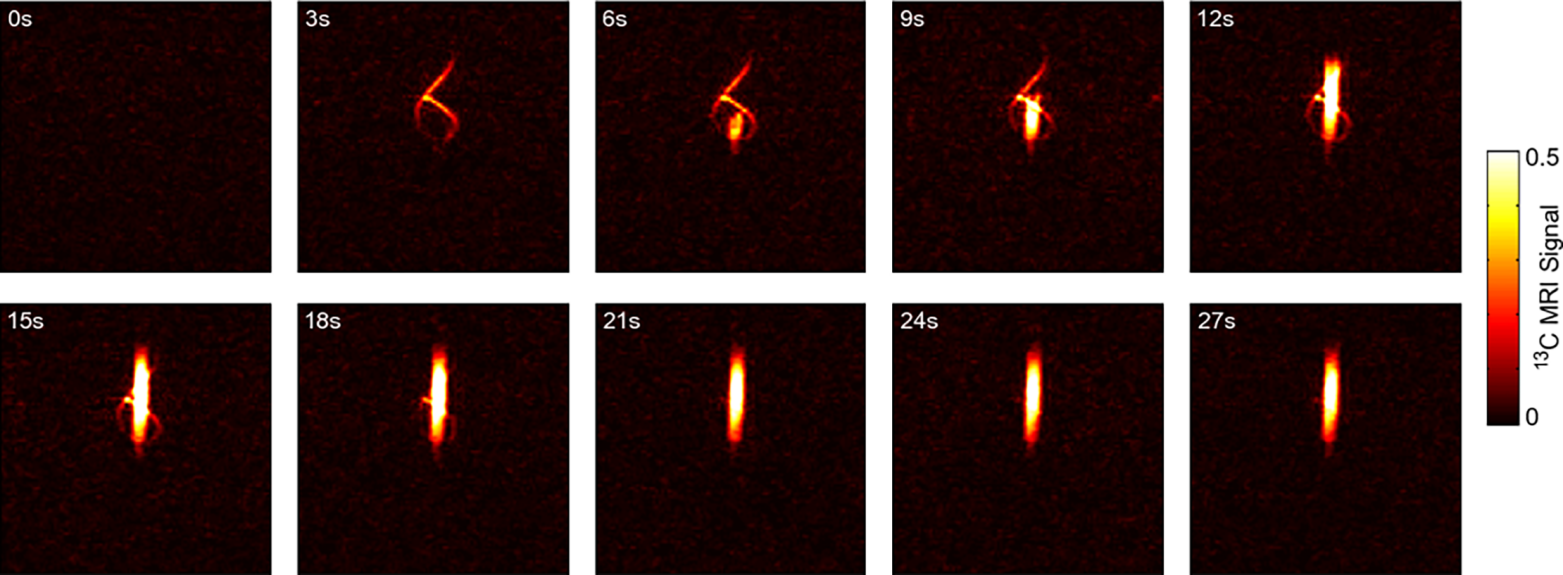
Dynamic ^13^C-MRI acquired during the injection of a SAMBADENA-produced agent. Ten images were recorded using Setup 2 with a repetition time of *T*_R_ = 3 s and an acquisition time of each image of ~500ms during the injection of ~ 600 μl tracer solution into a test object (HP solution: 5 mM HEA, 2 mM catalyst in H_2_O, *t*_h_ = 5 s; test object described in S4 Fig). Injection was started after the first scan and ended after the sixth (15s). Note that the injection was paused for MRI. After each scan, the magnetization was flipped back towards the longitudinal direction to conserve magnetization. The signal of all images was normalized with respect to the highest signal of image No. 6; the colour scale was trimmed to 0–0.5 of the maximum intensity to visualize the signal in the small hose. ^13^C-MRI parameters: 90/180°, RARE-factor: 38, FOV: (8.4cm)^2^, acquisition matrix of 96x96 px, interpolated to 256x256 px, in-plane resolution: 0.33 x 0.33 mm, one slice with a thickness of 6 cm, *T*_R_ = 0.487 s, *T*_E_ = 79 ms, acquisition time: 0.487 s.

In another experiment, a dynamic ^13^C-MRI was acquired during the injection of ~ 600 μl HP solution (5 mM HEA, 2 mM catalyst in H_2_O, *t*_h_ = 5 s). The imaging sequence was repeated back-to-back 10 times with a repetition time of *T*_R_ = 3 s (Fig 3). Here, to preserve signal, an additional pulse was added to each sequence to transform the transversal into longitudinal magnetization.

Note that in contrast to *in vivo* data all phantom images were acquired using Setup 2. The ^13^C-surface receive coil that was used in Setup 2 increased ^13^C-SNR by a factor 3 and 16.7 at a distance of 1.3 cm from the coil or adjacent to it, respectively (S2 and S3 Figs). Off course, the sensitive volume of the surface coil was smaller than that of the volume resonator.

### *In vivo* application

A solution containing ~ 80 mM HEP was polarized to ~ 4.9% within the MRI (80 mM HEA, 4.2 mM catalyst in H_2_O, *t*_h_ = 8 s). After the first and second injection from this batch, an axial or sagittal ^13^C-MRI was acquired, respectively, using Setup 1. Note that no flip-back pulse was applied after the first sequence such that the HP signal had mostly decayed before the second image was acquired. At no time did the tracer leave the magnet. The SNR of the ^13^C images was quantified to ~16 and 35 in the first and second scan, respectively.

**Fig 4:**
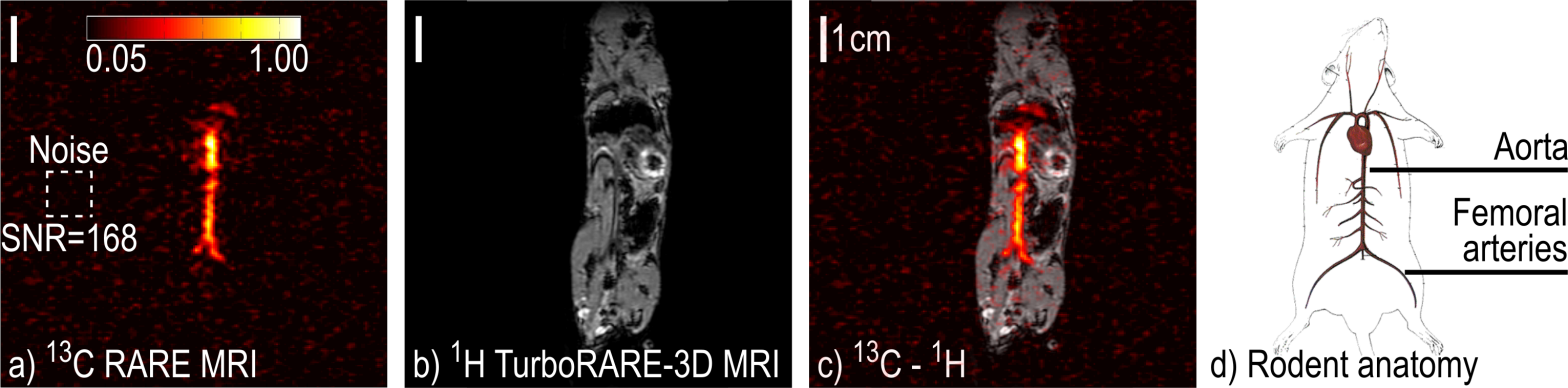
SAMBADENA HP of ^13^C HEP and subsequent ^13^C-MRI *in vivo*. ^13^C-MRI (a), ^1^H-MRI (b), ^1^H-^13^C co-registration (c) and schematic view (d) of a living mouse after the injection of ~80 mM HEP in 150 μl H_2_O (*P* ≈ 4% at the time of MRI). Strong ^13^C signal with an SNR of 35 was detected (a). A *T*_2_-weighted ^1^H-MRI (b) was acquired and co-registered with the ^13^C-image (c). The signal may be attributed to the *vena cava*, aorta and femoral arteries as shown schematically in (d). The isocenter of the magnet was the center of the images. Note that the tracer did not leave the magnet. [d): adapted with permission from www.biologycorner.com published under CC BY-NC-SA 4.0 license].

The most prominent signals were recorded in the second image and came from the *vena cava*, aorta and femoral arteries of the rodent (Fig 4 and S7 Fig). Because the catheter was filled with ~ 70–80 μl saline solution, only 70–80 μl of the first injection reached the animal. Likewise, before the second injection the catheter was still filled with the residues of the first and parts of it that were close to the resonator (maybe 10 – 20 μl) may have been depolarized by the first MRI.

The breathing rate and depth of the animal were monitored during and for 30 min after the injection of the combined dose of 300 μl, while the mouse was in the MRI. The animal did react only during the injections of the huge dose with a slightly slower and shallower respiration. After ~1 min no stress-related changes of the vital signs of the animal were observed.

## Discussion

In this contribution, the production, *in vivo* application and fast imaging of a SAMBADENA-polarized tracer was achieved. No critical and persistent effects on the vital signs of the animal were observed.

With the here-reported setup the production of one injection dose can be accomplished within a few seconds and the experiment can be repeated every ~2min once everything is set up and neglecting the preparation of the animal. More frequent injections appear feasible; however, concurrent HP and MRI is currently not possible.

Appropriate control of the temperature and pressure of the tracer before injection was identified to be a main challenge. With the method presented here, the pressure was successfully released from the reactor within 2 seconds, with losing less than 100 μl of HP solution. The temperature was found to drop rapidly to body temperatures due to heat exchange with the components of the injection system.

The entire setup was successfully realized in the narrow bore and the high magnetic field of the magnet. A smooth and steady injection of the tracer was achieved manually. Tracer and syringe (Fig 2) were kept inside the magnet. The syringe was easily reached from the back of the MRI scanner.

In the previous implementation [15], the reactor and animal had to be placed next to each other within the sensitive volume of the resonator (length of ~10 cm, diameter of 7.2 cm). Considering the dimensions of the current reactor with a length of 5.1 cm and a diameter of 7.1 cm, this approach left only little space for (parts of) a small rodent. Some parts were exceeding the homogeneous, sensitive volume of the resonator.

Here, this problem was circumvented by mounting reactor and animal bed together on an motorized slider. HP was performed with the reactor being in the isocenter of magnet and resonator. Then, during the temperature and pressure adjustment and the injection of the tracer solution, the slider moved the animal into the isocenter. In order to obtain a homogeneous magnetic field and accurate excitation fields (*B*_1_), adjustments in both positions were performed before the HP experiment and applied accordingly.

In this first attempt, however, the ^13^C frequency was not well adjusted to the ^13^C-resonance frequency of HEP. As Cartesian readout was performed, this issue resulted in a little spatial offset of the ^13^C-MRI from the ^1^H-MRI. ^13^C-frequency adjustment may be solved e.g., by a fast, automated frequency adjustment before ^13^C-MRI using a small fraction of the HP signal or by a more accurate adjustment prior to HP. Although further optimization is possible, the here described procedure was successfully applied, solving the afore-mentioned limitation of the method and enabling SAMBADENA for any animal that fits into the resonator.

In comparison to previous studies, where rats and pigs were imaged [31,32], mice are more challenging for angiographic ^13^C MRI, as the vessels–and thus required voxels–are smaller (typical aortic diameters of a 30 g mouse are ~1.2 mm [38]). Also the fast heart rate of mice of typically ~ 500 bpm (120 ms per cardiac cycle) makes a proper angiographic image acquisition more elaborate.

The current implementation leaves room for further improvement of HP and MRI. The latter by using a larger bolus (300 μl), optimizing the ^13^C-imaging sequence and using a ^13^C- surface receive coil as was already demonstrated *in vitro*. A faster and more complete hydrogenation reaction may further increase the HP. This could be achieved by optimizing the amount of dissolved *p*H_2_, reaction time, temperature and concentration of the tracer and catalyst as described in [15].

Currently, ^13^C-polarizations in excess of 20% were achieved for concentrations of the tracer HEP of ~ 5 mM. At a higher concentration of 80 mM, which is more favorable for *in vivo* application, the polarization decreases to *P* = (4.9 ± 0.6)%. We attribute this mostly to the fact that a smaller fraction of the precursor molecules is hydrogenated during the reaction. Note that the actual HP of all hydrogenated agents is expected to be mostly independent from its concentration. Interestingly, changing the concentration of catalyst in the range of *c*_cat_ = 1 mM – 4 mM did not change the polarization yield of a sample with 80 mM HEA. Increasing the *p*H_2_ pressure during the reaction to 30 bar, however, increased the HP from ~7% to ~13% previously [15]. Thus, a higher *p*H_2_ pressure is promising and currently being investigated.

It should be noted that almost no ^13^C signal from tracer inside the heart is seen in the acquired angiographic images. This might be explained by signal loss induced by the pronounced turbulent blood flow in the ventricles. Considering the average heart rate of ~ 500 bpm (120 ms per heart beat) and the image acquisition time of ~ 500 ms, the imaging signal is collected over the course of approximately four full cardiac cycles which may cause substantial dephasing because of turbulent flow and ultimately leads to signal loss. This effect is less prominent in the blood vessels which exhibit a more laminar and directional flow pattern so that dephasing effects are substantially smaller. The issue could be reduced, if data is recorded only during diastole of the cardiac cycle. Note that large blood flow velocities have been reported for mice e.g., up to 35cm/s in the pulmonary arteries during systole [39], whereas during diastole much smaller values, down to 0cm/s, are reached.

It is yet unclear, if hyperpolarized MR will be translated into clinical routine, although very promising human studies were undertaken in recent years [9,10]. While high cost and elaborate work-flow are a major hurdle of d-DNP, *p*H_2_-based methods like SAMBADENA are less expansive and complex. For PHIP methods, however, some significant challenges persist, namely a) obtaining a sterile, pure solution with no catalyst residuals, b) highly hyperpolarized, highly concentrated c) biologically relevant tracers. A recent overview is given in [40], and the main challenges are discussed below

- **Catalyst:** For human application, the hydrogenation catalyst must be removed before injection. The current catalysts are hazardous as they contain transition metals and organic compounds. For the catalyst used here, the used rhodium moiety is labeled with the hazard statement H314 (“Causes severe skin burns and eye damages”); toxicity, LD50 values or other hazard statements are, however, not reported, although some screening was performed [41]. The catalyst content may be reduced by adding an in-line filter in the injection path for homogeneous catalysts [33] or heterogeneous catalysts [26–29]. A biphasic approach was suggested where the catalyst-free, aqueous phase contains the HP agents. [23]. All of these methods, however, need to be improved and validated e.g. with respect to polarization yield. Another important issue is the sterility of the injection solution. Note that SAMBADENA-produced HP solutions were tested and certified as sterile previously [15].
- **Highly concentrated, highly polarized tracers:** *In vivo* imaging requires highly hyperpolarized, highly concentrated agents, e.g. *P* > 10% and *c* > 50 mM. d-DNP fulfills these conditions, but *p*H_2_-methods often report low polarization or low concentration. Part of this issue appears to be addressable by improving on the mechanics of the hydrogenation reaction, e.g. higher temperature and higher pressures.
- **Clinically relevant tracers**: The hitherto limited pool of agents amenable to *p*H_2_-HP was recently significantly extended by SABRE [42] and PHIP-SAH [24]. By now, molecules for receptor imaging [43,44], succinate [18,19,45], lactate [11–13,16], acetate [24] were polarized, and first metabolic studies were performed with PHIP-SAH-polarized pyruvate [11,24,46].
  Here, HEP was chosen for a proof-of-principle because of its high and robust polarization at low and high field [15,32–34,36,47,48], although there is no direct clinical relevance. HEA, the precursor of HEP, is known to be harmful at high concentrations and studies indicate oral LD_50_ values of 540–1070 mg/kg [49]; to our best knowledge, no such data exist for intravenous application. The application of 300 μl solution containing 80 mM HEP into a 30g mouse corresponds to 300 mg / kg and an in-blood-concentration of ~10 mM.

## Conclusions

*In vivo* ^13^C-MRI was demonstrated using an agent that was produced in the bore of the magnet seconds before imaging by SAMBADENA. For this method, a dedicated, complex and expensive external polarizer and the transfer of the agent into the MRI system is no longer needed.

After the first report in 2017 [15], this first *in vivo* demonstration is a key step towards metabolic MRI with this method, which holds the potential to become a fast, simple, low-cost alternative to produce HP tracers.

This development is particularly interesting in view of the recently extended portfolio of *p*H_2_-tracers, including pyruvate and acetate, (phospho-) lactate and succinate.

## Supporting information

S1 Text Quantification and improvement of hyperpolarization. (PDF)

S1 Fig Hyperpolarization as function of the concentration of the catalyst. HP experiments were repeated with different concentrations of the hydrogenation catalyst *in vitro* (concentration *c*_cat_ of 1 mM, 2 mM, 3 mM and 4 mM in H_2_O; concentration of substrate precursor *c*_HEA_ = 80 mM, temperature of *T* ≈ 80°C, *p*H_2_-pressure of *p* = 15 bar, hydrogenation time *t*_h_ = 8 s). No significant changes were observed and HP yields were equal within the error intervals. Each data point corresponds to the mean and standard error of *N* = 3 experiments. (*P*(1 mM) = (4.4 ± 0.3)%; *P*(2 mM) = (4.9 ± 0.3)%; *P*(3 mM) = (4.0 ± 0.8)%; *P*(4 mM) = (4.9 ± 0.6)%).
(PDF)

S2 Fig **(a) Axial** ^**1**^**H MRI of a** ^**13**^**C-enriched model solution** (Model solution M1, 3.3 mM 1-^13^C sodium acetate in 1.2 mL H_2_O). The surface loop coil was mounted to M1 as is indicated by a dashed line; the shape of M1 is outlined by a thin solid line. Region of interest (ROI) of signal and noise regions that were used for SNR quantification of scans (b) and (c) are represented by thick solid lines (S1 and S2: 1.25 x 1.66 mm, corresponding to 3 x 3 px in the ^13^C image; Noise: 8.31 x 11.08 mm corresponding to 20 x 20 px in the ^13^C image; MRI: single-shot RARE sequence, 90/180°, RARE factor 36, partial Fourier factor 1.7778, 128 x 64 matrix, FOV (6 cm)^2^, (0.47 x 0.94) mm^2^ in-plane resolution, 6-cm-slice thickness, TR = 3 s, TE = 15 ms, acquisition time 3 s, bandwidth 10 kHz, centred in the isocentre). **Axial** ^**13**^**C MRI of M1 recorded using either a** ^**1**^**H-**^**13**^**C transmit-receive volume resonator (b) or the** ^**13**^**C surface coil (c).** Note that in both cases the volume resonator was used for excitation, as described in the main article. SNR was quantified by dividing the average signal of ROI S1 or S2 (see (a)) by the standard deviation of the noise in the indicated ROI. S1 was adjacent to the coil and S2 at a distance of ~1.3 cm along the symmetry axis of the loop. Using the surface coil, the SNR1 and SNR2 (measured at S1 and S2, respectively) increased from 5.3 to 88.3 and 6.0 to 18.3, respectively, corresponding to a sensitivity enhancement factor of 16.7 or 3.0. Note that the surface coil was mounted on M1 in both images, but used for acquisition only in (c). All images were normalized to the highest signal in the corresponding image (MRI: single-shot RARE sequence, 90/180°, RARE factor 38, partial Fourier factor 1.7778, 128 x 96 matrix, FOV (8.4 cm)2, (0.85 x 0.64) mm^2^ in-plane resolution, 6-cm-slice thickness, TR = 0.487 s, TE = 79 ms, acquisition time 487 ms, bandwidth 10 kHz, centred in the isocentre).
(PDF)

S3 Fig **Non-localized** ^**13**^**C-NMR of M1 acquired with the volume coil (left) and with the surface coil (right).** The setup was as depicted in S2 Fig (a). The SNR was determined by dividing the highest signal intensity by the standard deviation of the noise in the region highlighted in the figures ((-10.36) – (-13.67) ppm). The SNR was quantified to 217 using the volume resonator and increased to 2320 using the surface coil for data acquisition. Thus, the SNR over the total sample volume of M1 was increased by a factor of 10.7 when using the surface coil.
(PDF)

S4 Fig An imaging-test object (phantom) for optimizing the injection and MRI. A tube with an inner diameter of 1mm was wound around a 1ml syringe and one end of the hose was connected to the tip of the syringe. Both were centred in a 15 ml Falcon tube, which was filled with deionized H_2_O (see right in the figure). The handle of the syringe was pushed out by an injected solution. After the experiment, the phantom was actuated manually to empty the phantom. The other end of the phantom-tube was connected to the injection setup described in the main article. During experiments the phantom was placed on the mouse bed and the injection syringe was connected to the reactor (see left in the image). The image field of view (FOV) that was used in S5 and S6 Figs and Fig 3 of the main article is indicated on the right.
(PDF)

S5 Fig *In vitro* MRI with SAMBADENA-produced tracer solution. The phantom was mounted to the setup as described in S4 Fig and an HP experiment was performed (*c*_HEA_ = 5 mM, *c*_cat_ = 1 mM, *t*_hyd_ = 5 s, 80°C, 15 bar *p*H_2_). The tracer was extracted from the reactor and 150μl were injected into the phantom, similarly to the *in vivo* experiment described in the main text. Subsequently, ^13^C-MRI was acquired ((a), co-registrated with an ^1^H-MRI (b), which depicted the tracer solution in the tube of the phantom (see S4 Fig) (c). Note that in contrast to *in vivo* experiments, ^13^C was recorded using a ^13^C-surface coil (S2 Fig). Both, ^1^H- and ^13^C-MRI were normalized to the highest signal in the image. A 5 mM tracer solution was chosen because concentration and magnetization are similar to *in vivo* experiments (300μl, 80 mM tracer dilute with ~ 2.1ml blood of a 30g mouse and yield a final tracer concentration of ~10 mM *in vivo*; additionally, as HP of a concentrated sample is lower (see Supplementary Information 1), a 2x lower concentration was chosen). ^13^C-MRI: 90/180°, RARE-factor: 38, FOV: (8.4cm)^2^, acquisition matrix of 128x96 px, interpolated to 256x256 px, in-plane resolution: 0.33 x 0.33 mm, one slice with thickness: 6 cm, *T*_R_ = 0.487 s, *T*_E_ = 79 ms, acquisition time: 0.487 s.
(PDF)

S6 Fig Reproducibility of ^13^C-MRI and SNR. The experiment from S5 Fig was repeated three times and 300μl tracer solution were injected each time. Using the indicated noise region (right) the signal to noise ratio (SNR) was determined in each image at two positions: (a) using the highest signal in the image (SNRmax) and (b) using a selected region in the catheter, indicated as S1 in the centre image (SNRcat). The mean values with corresponding standard deviation of the three scans were determined to SNR1 = (418 ± 72) and SNR2 = (110 ± 29).
(PDF)

S7 Fig ^13^C-MRI of SAMBADENA-polarized hydroxyethyl-propionate *in vivo*. One hyperpolarized batch of 600 μl was used for two separate injections (each 150 μl) into a 30g mouse via the tail-vein (HP of *P*≈5%, *c*_HEA_ = 80mM, *c*_cat_ = 4mM; temperature of *T* = (35±1)°C). After each injection ^13^C-MRI was acquired: first an axial image, 15s after HP with a signal to noise ratio of SNR1 = 16 (left); next a sagittal image, 30s after HP with SNR2 = 35 (right). SNR was quantified as highest signal in the image divided by standard deviation of the noise in the indicated region. SNR of the first image was lower, because the catheter was filled with ~ 70–80 μl saline solution before experiments and thus, only 70–80 μl of the first injection reached the animal (also see main text). Noise of both images was matched and signal intensities were normalized to the highest signal of the second scan. ^13^C-MRI sequence: 90/180°, RARE-factor: 38, FOV: (8.4cm)^2^, acquisition matrix of 128x96 px, interpolated to 128x128 px, in-plane resolution: 0.66 x 0.66 mm, one slice with thickness: 6 cm, *T*_R_ = 0.487 s, *T*_E_ = 79 ms, acquisition time: 0.487 s.
(PDF)

S1 Dataset Data and protocol. A compressed file containing data reported in this article along with a lab protocol.
(RAR)

S2 Dataset *In vivo* data. A compressed file containing in vivo data reported in this article.
(RAR)
