# Supplementary material for: In vivo 13C-MRI using SAMBADENA
Source: PLoS One. 2018 Jul 12;13(7):e0200141. doi: 10.1371/journal.pone.0200141 (PMC6042716; doi:10.1371/journal.pone.0200141)
Supplement: S1 Text — (PDF) [file pone.0200141.s001.pdf]

In vivo 13C-MRI using SAMBADENA

S1 Text

Quantification and Improvement of Hyperpolarization

The absolute ^{13}C polarization P of a hyperpolarized sample was quantified *in vitro* with respect to the signal of a thermally polarized sample that was acquired in a different scan (5ml acetone held in a test tube; natural abundance of ^{13}C of 1.1%; carbonyl-group). The following formula was used:

$$P = P_{\text{therm}} \cdot \frac{N_{\text{ref}}}{N_{\text{HP}}} \frac{F_{\text{13C-ref}}}{F_{\text{13C-HP}}} \frac{c_{\text{ref}}}{c_{\text{HP}}} \frac{V_{\text{ref}}}{V_{\text{HP}}} \frac{S_{\text{HP}}}{S_{\text{ref}}} \quad \text{(E. 1)}$$

'HP' and 'ref' indicate values of the hyperpolarized sample and the reference model solution, respectively. N is the number of summated scans (1 for all HP experiments), F_{13C} is the atomic fraction of the ^{13}C isotope, c is the concentration of the molecule, V is the volume of the sample and S the measured ^{13}C-NMR signal. The thermal polarization of ^{13}C at 7T is $P_{\text{therm}} \approx$ 6 ppm.

The signals were numerically integrated after multiplication with an exponential function (10 Hz), Fourier-transformation and automated phase and baseline correction (Topspin 2.0, Bruker, Germany).

Note that the hyperpolarization (HP) that was achieved in this contribution was ~5% for an 80mM sample. However, for a 5mM sample HP of P>20% were reported previously [1]. This matter is discussed in more detail in the following:

(a) **Quantification of Hyperpolarization:** The reported hyperpolarization values always assume a 100% hydrogenation. Thus, if less hydrogenation was achieved – which is rather the case for a highly concentrated tracer –, the reported polarization was lower than the actual polarization, which refers to hydrogenated molecules only.
The same applies to the enrichment of *para*hydrogen, which was assumed to be 100%. The bottom line is that there is space for further optimization by increasing the hydrogenated fraction.

(b) **Measuring the hydrogenated fraction:** The hydrogenation couldn't be measured using the MRI system as sensitivity and/or spectral resolution were insufficient. Moving to a higher field (400MHz) and higher substrate concentrations (100mM) did not solve the issue. Samples taken after the hyperpolarization experiment exhibited 100% hydrogenation (measured by high-resolution NMR). However, we found that hydrogenation was not completed after typical hydrogenation times (4-8 s) but ongoing after the hyperpolarization experiment as discussed next.

(c) **Model approach:** Thus, in the first report [1] (ref. [14] of the main article) a model was derived and fitted to experimental data where HP was measured as function of the hydrogenation time. From the model, the hydrogenation time constant and the relaxation time of the pH_2 spin order in the molecule were extracted. This data suggests that after a hydrogenation time of 8s, approx. 99 % of the precursor molecules of a 5mM tracer solution are hydrogenated and yield an HP of P~19% (P~19% after hydrogenation time of t_h=8s; P~21% after t_h=5s where 85% of the precursor was hydrogenated but less relaxation had occurred; for the latter the real polarization increases to 25% of the hydrogenated molecules instead of 21% considering all molecules (precursor and product)).
We consider the efficiency of the Spin Order Transfer and the relaxation of the *para*-H_2 spin-order being

independent of the concentration of the tracer. Hence, less complete hydrogenation is assumed to be the main reason for lower average polarization. Considering tracer concentrations of 5mM and 80mM and polarizations of 19% and 5%, respectively, the data suggests that for the latter only ~25% of the precursor was hydrogenated, leaving much space for improvement. Note that magnetization (~concentration*polarization) still was larger at 80mM by a factor of 4.2 $((5\% \cdot 80mM)/(19\% \cdot 5mM))$.

(d) **The way to go:** It is concluded that HP can be increased along with the efficiency of the hydrogenation (temperature, pressure, pH_2 concentration/distribution in solution, efficiency of the catalyst). Changing the concentration of the catalyst in the range of 1mM to 4mM did not change the polarization yield significantly (S1 Fig). Previously, however, HP of an 80mM sample was increased from ~7% to ~13% by increasing the pH_2-pressure from 15 to 30bar ([1], Supplementary Information). A new setup for higher pressures is currently being investigated. In addition, the reactor should be modified to minimize the size of the pH_2-bubbles and, thus, increase the surface to dissolve pH_2.

Reference:

1. Schmidt AB, Berner S, Schimpf W, Müller C, Lickert T, Schwaderlapp N, et al. Liquid-state carbon-13 hyperpolarization generated in an MRI system for fast imaging. Nat Commun. 2017; doi:10.1038/ncomms14535
