# Supplementary material for: In vivo 13C-MRI using SAMBADENA
Source: PLoS One. 2018 Jul 12;13(7):e0200141. doi: 10.1371/journal.pone.0200141 (PMC6042716; doi:10.1371/journal.pone.0200141)
Supplement: S1 Fig — HP experiments were repeated with different concentrations of the hydrogenation catalyst in vitro (concentration ccat of 1 mM, 2 mM, 3 mM and 4 mM in H2O; concentration of substrate precursor cHEA = 80 mM, temperature of T ≈ 80°C, pH2-pressure of p = 15 bar, hydrogenation time th = 8 s). No significant changes were observed and HP yields were equal within the error intervals. Each data point corresponds to the mean and standard error of N = 3 experiments. (P(1 mM) = (4.4 ± 0.3)%; P(2 mM) = (4.9 ± 0.3)%; P(3 mM) = (4.0 ± 0.8)%; P(4 mM) = (4.9 ± 0.6)%). (PDF) [file pone.0200141.s002.pdf]

In vivo 13C-MRI using SAMBADENA
S1 Fig

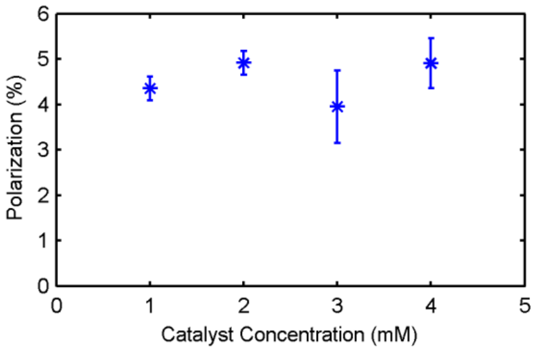

S1 Fig: Hyperpolarization as function of the concentration of the catalyst: HP experiments were repeated with different concentrations of the hydrogenation catalyst *in vitro* (concentration c_{cat} of 1 mM, 2 mM, 3 mM and 4 mM in H_2O; concentration of substrate precursor c_{HEA} = 80 mM, temperature of $T \approx 80$ °C, pH_2-pressure of p = 15 bar, hydrogenation time t_h = 8 s). No significant changes were observed and HP yields were equal within the error intervals. Each data point corresponds to the mean and standard error of N = 3 experiments.
(P(1 mM) = (4.4 ± 0.3) % ; P(2 mM) = (4.9 ± 0.3) % ; P(3 mM) = (4.0 ± 0.8) % ; P(4 mM) = (4.9 ± 0.6) %).
