# Supplementary material for: In vivo 13C-MRI using SAMBADENA
Source: PLoS One. 2018 Jul 12;13(7):e0200141. doi: 10.1371/journal.pone.0200141 (PMC6042716; doi:10.1371/journal.pone.0200141)
Supplement: S2 Fig — (a) Axial 1H MRI of a 13C-enriched model solution (Model solution M1, 3.3 mM 1-13C sodium acetate in 1.2 mL H2O). The surface loop coil was mounted to M1 as is indicated by a dashed line; the shape of M1 is outlined by a thin solid line. Region of interest (ROI) of signal and noise regions that were used for SNR quantification of scans (b) and (c) are represented by thick solid lines (S1 and S2: 1.25 x 1.66 mm, corresponding to 3 x 3 px in the 13C image; Noise: 8.31 x 11.08 mm corresponding to 20 x 20 px in the 13C image; MRI: single-shot RARE sequence, 90/180°, RARE factor 36, partial Fourier factor 1.7778, 128 x 64 matrix, FOV (6 cm)2, (0.47 x 0.94) mm2 in-plane resolution, 6-cm-slice thickness, TR = 3 s, TE = 15 ms, acquisition time 3 s, bandwidth 10 kHz, centred in the isocentre). Axial 13C MRI of M1 recorded using either a 1H-13C transmit-receive volume resonator (b) or the 13C surface coil (c). Note that in both cases the volume resonator was used for excitation, as described in the main article. SNR was quantified by dividing the average signal of ROI S1 or S2 (see (a)) by the standard deviation of the noise in the indicated ROI. S1 was adjacent to the coil and S2 at a distance of ~1.3 cm along the symmetry axis of the loop. Using the surface coil, the SNR1 and SNR2 (measured at S1 and S2, respectively) increased from 5.3 to 88.3 and 6.0 to 18.3, respectively, corresponding to a sensitivity enhancement factor of 16.7 or 3.0. Note that the surface coil was mounted on M1 in both images, but used for acquisition only in (c). All images were normalized to the highest signal in the corresponding image (MRI: single-shot RARE sequence, 90/180°, RARE factor 38, partial Fourier factor 1.7778, 128 x 96 matrix, FOV (8.4 cm)2, (0.85 x 0.64) mm2 in-plane resolution, 6-cm-slice thickness, TR = 0.487 s, TE = 79 ms, acquisition time 487 ms, bandwidth 10 kHz, centred in the isocentre). (PDF) [file pone.0200141.s003.pdf]

In vivo 13C-MRI using SAMBADENA

S2 Fig

Improving SNR with a ^{13}C Receive Surface Coil

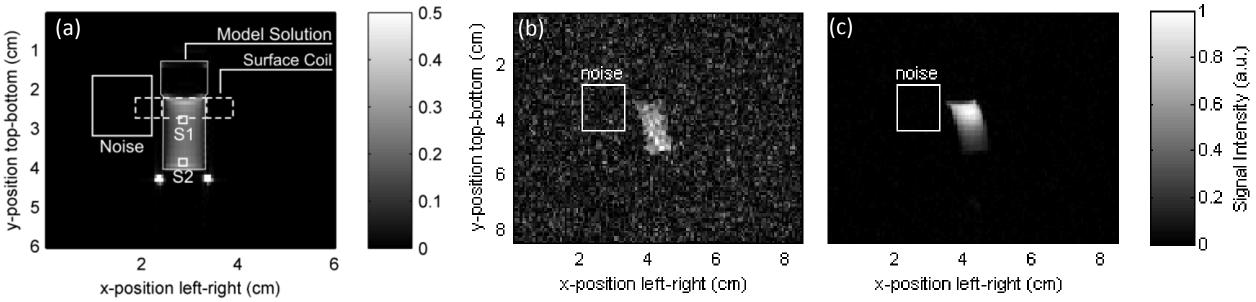

S2 Fig: **(a) Axial ^{1}H MRI of a ^{13}C-enriched model solution** (Model solution M1, 3.3 mM 1-^{13}C sodium acetate in 1.2 mL H_2O). The surface loop coil was mounted to M1 as is indicated by a dashed line; the shape of M1 is outlined by a thin solid line. Region of interest (ROI) of signal and noise regions that were used for SNR quantification of scans (b) and (c) are represented by thick solid lines (S1 and S2: 1.25 x 1.66 mm, corresponding to 3 x 3 px in the ^{13}C image; Noise: 8.31 x 11.08 mm corresponding to 20 x 20 px in the ^{13}C image; MRI: single-shot RARE sequence, 90/180°, RARE factor 36, partial Fourier factor 1.7778, 128 x 64 matrix, FOV (6 cm)^2, (0.47 x 0.94) mm^2 in-plane resolution, 6-cm-slice thickness, TR = 3 s, TE = 15 ms, acquisition time 3 s, bandwidth 10 kHz, centred in the isocentre).

Axial ^{13}C MRI of M1 recorded using either a ^{1}H-^{13}C transmit-receive volume resonator (b) or the ^{13}C surface coil (c). Note that in both cases the volume resonator was used for excitation, as described in the main article. SNR was quantified by dividing the average signal of ROI S1 or S2 (see (a)) by the standard deviation of the noise in the indicated ROI. S1 was adjacent to the coil and S2 at a distance of ~1.3 cm along the symmetry axis of the loop. Using the surface coil, the SNR1 and SNR2 (measured at S1 and S2, respectively) increased from 5.3 to 88.3 and 6.0 to 18.3, respectively, corresponding to a sensitivity enhancement factor of 16.7 or 3.0. Note that the surface coil was mounted on M1 in both images, but used for acquisition only in (c). All images were normalized to the highest signal in the corresponding image (MRI: single-shot RARE sequence, 90/180°, RARE factor 38, partial Fourier factor 1.7778, 128 x 96 matrix, FOV (8.4 cm)2, (0.85 x 0.64) mm^2 in-plane resolution, 6-cm-slice thickness, TR = 0.487 s, TE = 79 ms, acquisition time 487 ms, bandwidth 10 kHz, centred in the isocentre).
