# Supplementary material for: In vivo 13C-MRI using SAMBADENA
Source: PLoS One. 2018 Jul 12;13(7):e0200141. doi: 10.1371/journal.pone.0200141 (PMC6042716; doi:10.1371/journal.pone.0200141)
Supplement: S3 Fig — Non-localized 13C-NMR of M1 acquired with the volume coil (left) and with the surface coil (right). The setup was as depicted in S2 Fig (a). The SNR was determined by dividing the highest signal intensity by the standard deviation of the noise in the region highlighted in the figures ((-10.36) – (-13.67) ppm). The SNR was quantified to 217 using the volume resonator and increased to 2320 using the surface coil for data acquisition. Thus, the SNR over the total sample volume of M1 was increased by a factor of 10.7 when using the surface coil. (PDF) [file pone.0200141.s004.pdf]

In vivo 13C-MRI using SAMBADENA

S3 Fig

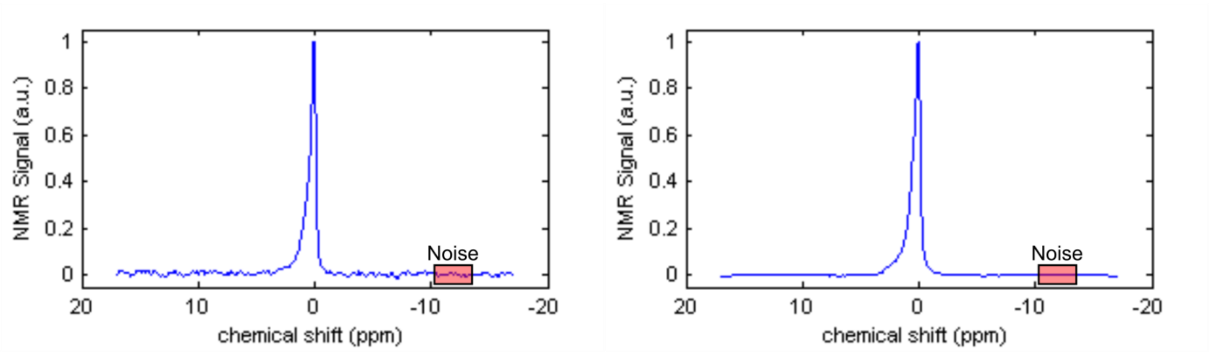

S3 Fig: Non-localized ^{13}C-NMR of M1 acquired with the volume coil (left) and with the surface coil (right). The setup was as depicted in S2 Fig (a). The SNR was determined by dividing the highest signal intensity by the standard deviation of the noise in the region highlighted in the figures ((-10.36) – (-13.67) ppm). The SNR was quantified to 217 using the volume resonator and increased to 2320 using the surface coil for data acquisition. Thus, the SNR over the total sample volume of M1 was increased by a factor of 10.7 when using the surface coil.
