# Supplementary material for: In vivo 13C-MRI using SAMBADENA
Source: PLoS One. 2018 Jul 12;13(7):e0200141. doi: 10.1371/journal.pone.0200141 (PMC6042716; doi:10.1371/journal.pone.0200141)
Supplement: S4 Fig — A tube with an inner diameter of 1mm was wound around a 1ml syringe and one end of the hose was connected to the tip of the syringe. Both were centred in a 15 ml Falcon tube, which was filled with deionized H2O (see right in the figure). The handle of the syringe was pushed out by an injected solution. After the experiment, the phantom was actuated manually to empty the phantom. The other end of the phantom-tube was connected to the injection setup described in the main article. During experiments the phantom was placed on the mouse bed and the injection syringe was connected to the reactor (see left in the image). The image field of view (FOV) that was used in S5 and S6 Figs and Fig 3 of the main article is indicated on the right. (PDF) [file pone.0200141.s005.pdf]

In vivo 13C-MRI using SAMBADENA

S4 Fig

Imaging Test Object for Injection and ^{13}C-MRI

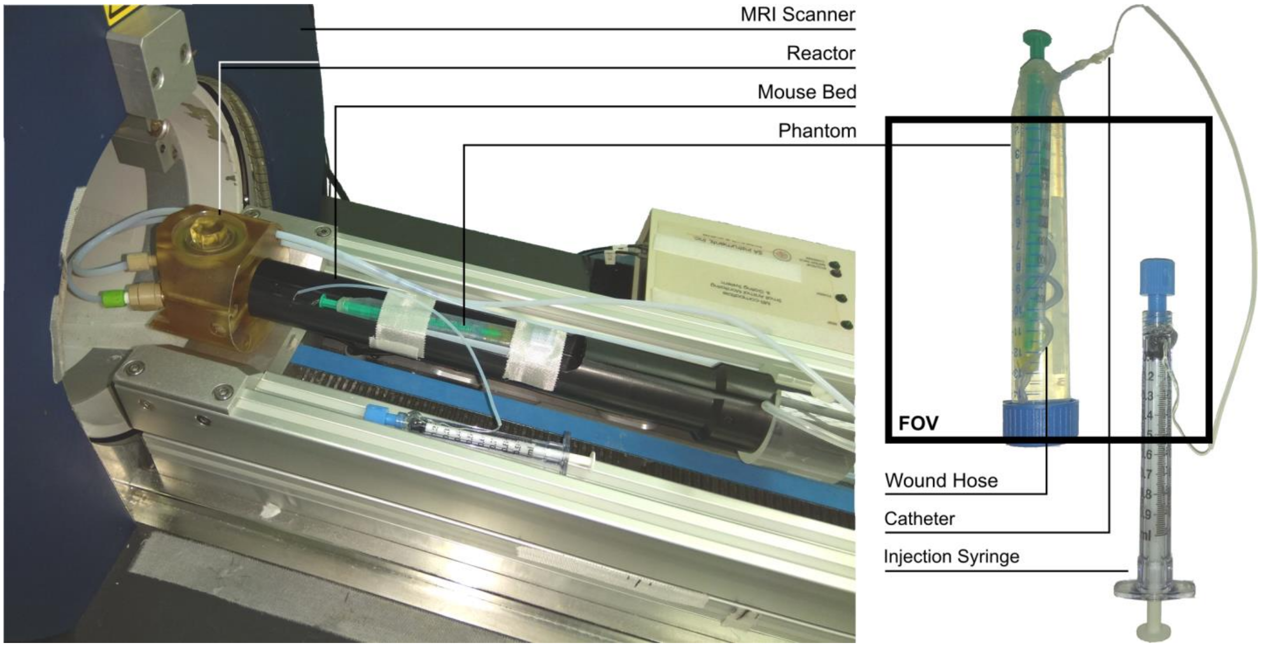

S4 Fig: An imaging-test object (phantom) for optimizing the injection and MRI. A tube with an inner diameter of 1mm was wound around a 1ml syringe and one end of the hose was connected to the tip of the syringe. Both were centred in a 15 ml Falcon tube, which was filled with deionized H_2O (see right in the figure). The handle of the syringe was pushed out by an injected solution. After the experiment, the phantom was actuated manually to empty the phantom. The other end of the phantom-tube was connected to the injection setup described in the main article. During experiments the phantom was placed on the mouse bed and the injection syringe was connected to the reactor (see left in the image). The image field of view (FOV) that was used in S5 and S6 Figs and Fig. 3 of the main article is indicated on the right.
