# Supplementary material for: In vivo 13C-MRI using SAMBADENA
Source: PLoS One. 2018 Jul 12;13(7):e0200141. doi: 10.1371/journal.pone.0200141 (PMC6042716; doi:10.1371/journal.pone.0200141)
Supplement: S5 Fig — The phantom was mounted to the setup as described in S4 Fig and an HP experiment was performed (cHEA = 5 mM, ccat = 1 mM, thyd = 5 s, 80°C, 15 bar pH2). The tracer was extracted from the reactor and 150μl were injected into the phantom, similarly to the in vivo experiment described in the main text. Subsequently, 13C-MRI was acquired ((a), co-registrated with an 1H-MRI (b), which depicted the tracer solution in the tube of the phantom (see S4 Fig) (c). Note that in contrast to in vivo experiments, 13C was recorded using a 13C-surface coil (S2 Fig). Both, 1H- and 13C-MRI were normalized to the highest signal in the image. A 5 mM tracer solution was chosen because concentration and magnetization are similar to in vivo experiments (300μl, 80 mM tracer dilute with ~ 2.1ml blood of a 30g mouse and yield a final tracer concentration of ~10 mM in vivo; additionally, as HP of a concentrated sample is lower (see Supplementary Information 1), a 2x lower concentration was chosen). 13C-MRI: 90/180°, RARE-factor: 38, FOV: (8.4cm)2, acquisition matrix of 128x96 px, interpolated to 256x256 px, in-plane resolution: 0.33 x 0.33 mm, one slice with thickness: 6 cm, TR = 0.487 s, TE = 79 ms, acquisition time: 0.487 s. (PDF) [file pone.0200141.s006.pdf]

In vivo 13C-MRI using SAMBADENA

S5 Fig

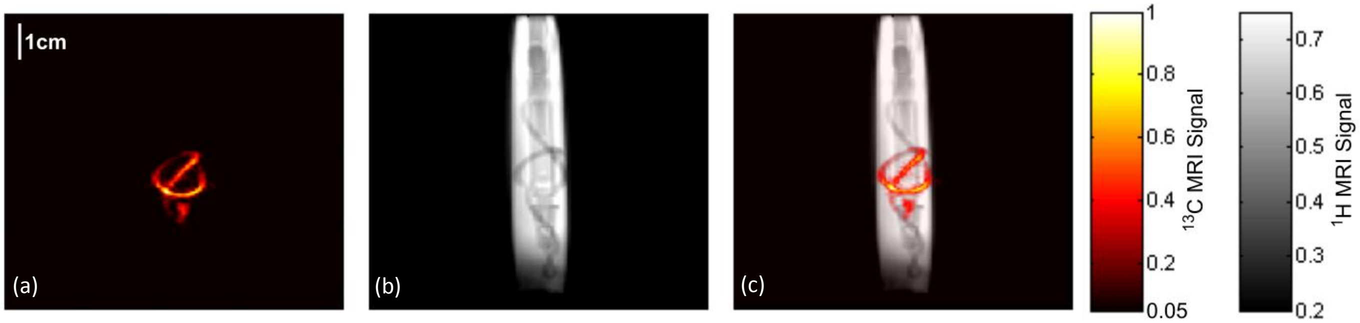

S5 Fig: *In vitro* MRI with SAMBADENA-produced tracer solution. The phantom was mounted to the setup as described in S4 Fig and an HP experiment was performed (c_{HEA} = 5 mM, c_{cat} = 1 mM, t_{hyd} = 5 s, 80 °C, 15 bar pH_2). The tracer was extracted from the reactor and 150µl were injected into the phantom, similarly to the *in vivo* experiment described in the main text. Subsequently, ^{13}C-MRI was acquired ((a), co-registrated with an ^{1}H-MRI (b), which depicted the tracer solution in the tube of the phantom (see S4 Fig) (c). Note that in contrast to *in vivo* experiments, ^{13}C was recorded using a ^{13}C-surface coil (S2 Fig). Both, ^{1}H- and ^{13}C-MRI were normalized to the highest signal in the image. A 5 mM tracer solution was chosen because concentration and magnetization are similar to *in vivo* experiments (300µl, 80 mM tracer dilute with ~ 2.1ml blood of a 30g mouse and yield a final tracer concentration of ~10 mM *in vivo*; additionally, as HP of a concentrated sample is lower (see Supplementary Information 1), a 2x lower concentration was chosen). ^{13}C-MRI: 90/180°, RARE-factor: 38, FOV: (8.4cm)^2, acquisition matrix of 128x96 px, interpolated to 256x256 px, in-plane resolution: 0.33 x 0.33 mm, one slice with thickness: 6 cm, T_R = 0.487 s, T_E = 79 ms, acquisition time: 0.487 s**.**
