# Supplementary material for: In vivo 13C-MRI using SAMBADENA
Source: PLoS One. 2018 Jul 12;13(7):e0200141. doi: 10.1371/journal.pone.0200141 (PMC6042716; doi:10.1371/journal.pone.0200141)
Supplement: S6 Fig — The experiment from S5 Fig was repeated three times and 300μl tracer solution were injected each time. Using the indicated noise region (right) the signal to noise ratio (SNR) was determined in each image at two positions: (a) using the highest signal in the image (SNRmax) and (b) using a selected region in the catheter, indicated as S1 in the centre image (SNRcat). The mean values with corresponding standard deviation of the three scans were determined to SNR1 = (418 ± 72) and SNR2 = (110 ± 29). (PDF) [file pone.0200141.s007.pdf]

In vivo 13C-MRI using SAMBADENA

S6 Fig

Reproducibility of SNR

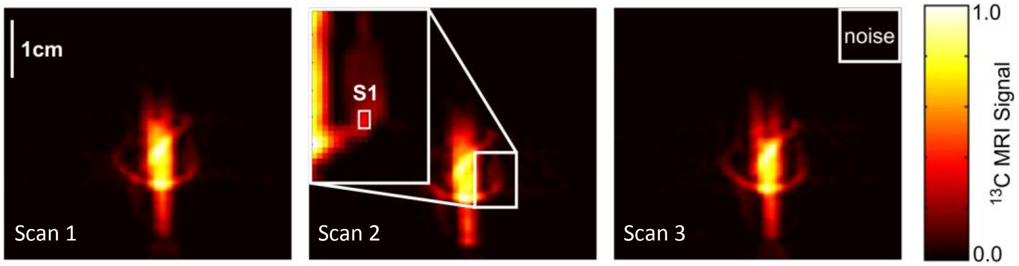

S6 Fig: Reproducibility of ^{13}C-MRI and SNR. The experiment from S5 Fig was repeated three times and 300µl tracer solution were injected each time. Using the indicated noise region (right) the signal to noise ratio (SNR) was determined in each image at two positions: (a) using the highest signal in the image (SNRmax) and (b) using a selected region in the catheter, indicated as S1 in the centre image (SNRcat). The mean values with corresponding standard deviation of the three scans were determined to SNR1 = (418 ± 72) and SNR2 = (110 ± 29).
