# Supplementary material for: In vivo 13C-MRI using SAMBADENA
Source: PLoS One. 2018 Jul 12;13(7):e0200141. doi: 10.1371/journal.pone.0200141 (PMC6042716; doi:10.1371/journal.pone.0200141)
Supplement: S7 Fig — One hyperpolarized batch of 600 μl was used for two separate injections (each 150 μl) into a 30g mouse via the tail-vein (HP of P≈5%, cHEA = 80mM, ccat = 4mM; temperature of T = (35±1)°C). After each injection 13C-MRI was acquired: first an axial image, 15s after HP with a signal to noise ratio of SNR1 = 16 (left); next a sagittal image, 30s after HP with SNR2 = 35 (right). SNR was quantified as highest signal in the image divided by standard deviation of the noise in the indicated region. SNR of the first image was lower, because the catheter was filled with ~ 70–80 μl saline solution before experiments and thus, only 70–80 μl of the first injection reached the animal (also see main text). Noise of both images was matched and signal intensities were normalized to the highest signal of the second scan. 13C-MRI sequence: 90/180°, RARE-factor: 38, FOV: (8.4cm)2, acquisition matrix of 128x96 px, interpolated to 128x128 px, in-plane resolution: 0.66 x 0.66 mm, one slice with thickness: 6 cm, TR = 0.487 s, TE = 79 ms, acquisition time: 0.487 s. (PDF) [file pone.0200141.s008.pdf]

In vivo 13C-MRI using SAMBADENA

S7 Fig

In vivo MRI

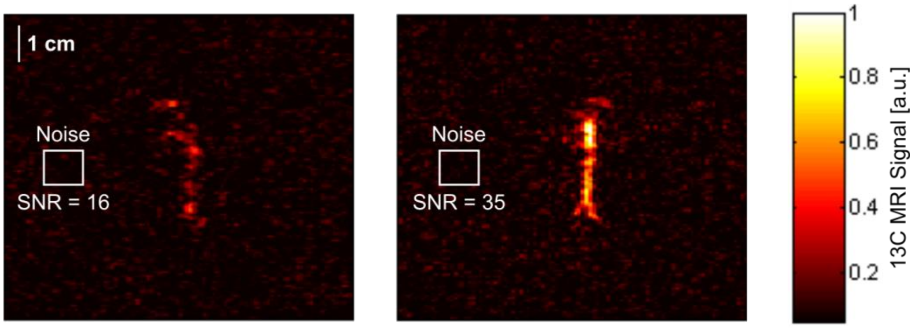

S7 Fig: ^{13}C-MRI of SAMBADENA-polarized hydroxyethyl-propionate *in vivo*. One hyperpolarized batch of 600 µl was used for two separate injections (each 150 µl) into a 30g mouse via the tail-vein (HP of P≈5%, c_{HEA} = 80mM, c_{cat} = 4mM; temperature of T = (35±1) °C). After each injection ^{13}C-MRI was acquired: first an axial image, 15s after HP with a signal to noise ratio of SNR1=16 (left); next a sagittal image, 30s after HP with SNR2=35 (right). SNR was quantified as highest signal in the image divided by standard deviation of the noise in the indicated region. SNR of the first image was lower, because the catheter was filled with ~ 70 – 80 µl saline solution before experiments and thus, only 70 – 80 µl of the first injection reached the animal (also see main text). Noise of both images was matched and signal intensities were normalized to the highest signal of the second scan. ^{13}C-MRI sequence: 90/180°, RARE-factor: 38, FOV: (8.4cm)^2, acquisition matrix of 128x96 px, interpolated to 128x128 px, in-plane resolution: 0.66 x 0.66 mm, one slice with thickness: 6 cm, T_R = 0.487 s, T_E = 79 ms, acquisition time: 0.487 s.
